# Enhanced Deep Learning Model for Classification of Retinal Optical Coherence Tomography Images

**DOI:** 10.3390/s23125393

**Published:** 2023-06-07

**Authors:** Esraa Hassan, Samir Elmougy, Mai R. Ibraheem, M. Shamim Hossain, Khalid AlMutib, Ahmed Ghoneim, Salman A. AlQahtani, Fatma M. Talaat

**Affiliations:** 1 Faculty of Artificial Intelligence, Kafrelsheikh University, Kafrelsheikh 33516, Egypt; esraa.hassan@ai.kfs.edu.eg (E.H.); fatma.nada@ai.kfs.edu.eg (F.M.T.); 2 Department of Computer Science, Faculty of Computers and Information, Mansoura University, Mansoura 35516, Egypt; mougy@mans.edu.eg; 3 Department of Information Technology, Faculty of Computers and information, Kafrelsheikh University, Kafrelsheikh 33516, Egypt; mai_amer@fci.kfs.edu.eg; 4 Research Chair of Pervasive and Mobile Computing, Department of Software Engineering, College of Computer and Information Sciences, King Saud University, Riyadh 11543, Saudi Arabia; ghoneim@ksu.edu.sa; 5 Department of Software Engineering, College of Computer and Information Sciences, King Saud University, Riyadh 11574, Saudi Arabia; muteb@ksu.edu.sa; 6 Research Chair of Pervasive and Mobile Computing, Department of Computer Engineering, College of Computer and Information Sciences, King Saud University, Riyadh 11574, Saudi Arabia; salmanq@ksu.edu.sa

**Keywords:** optical coherence tomography (OCT), deep learning, artificial intelligence, optical sensor technologies

## Abstract

Retinal optical coherence tomography (OCT) imaging is a valuable tool for assessing the condition of the back part of the eye. The condition has a great effect on the specificity of diagnosis, the monitoring of many physiological and pathological procedures, and the response and evaluation of therapeutic effectiveness in various fields of clinical practices, including primary eye diseases and systemic diseases such as diabetes. Therefore, precise diagnosis, classification, and automated image analysis models are crucial. In this paper, we propose an enhanced optical coherence tomography (EOCT) model to classify retinal OCT based on modified ResNet (50) and random forest algorithms, which are used in the proposed study’s training strategy to enhance performance. The Adam optimizer is applied during the training process to increase the efficiency of the ResNet (50) model compared with the common pre-trained models, such as spatial separable convolutions and visual geometry group (VGG) (16). The experimentation results show that the sensitivity, specificity, precision, negative predictive value, false discovery rate, false negative rate accuracy, and Matthew’s correlation coefficient are 0.9836, 0.9615, 0.9740, 0.9756, 0.0385, 0.0260, 0.0164, 0.9747, 0.9788, and 0.9474, respectively.

## 1. Introduction

As a subsurface imaging modality with multiple biomedical and industrial uses, OCT has become increasingly popular in recent years in various fundamental research areas, such as lasers, optical sensors, and sparse signal processing. OCT provides high-resolution cross-sectional images of the ocular tissue and is now often used in clinical care practice and research to obtain a better understanding of the eye in both healthy instances and during illness; it has revolutionized the imaging of the eye [1]. The quantification of the acquired images is necessary to better understand the eye’s normal development and the impact of common eye conditions such as myopia upon eye morphology; it is also necessary to facilitate early disease detection. OCT images can be used to guide clinical decisions such as monitoring, detecting, classifying, and managing eye health and diseases, helping to monitor the current treatment of eye diseases and diagnose eye diseases early.

Swept-source OCT technology, such as the IOL Master 700 and Eyestar 900 systems, is used in ocular biometry to measure various ocular parameters such as axial length and anterior chamber depth. These measurements can be useful in evaluating changes in these parameters, particularly in cases of pathological myopia. The study in [2] aimed to investigate the influence of changes in axial length (AL) after cataract surgery on the intraocular lens (IOL) power calculation. The patients underwent an ophthalmic evaluation before surgery and two months after surgery. An optical biometry was performed using the IOL Master 500, and the same measurements were repeated after the surgery. The changes in the AL and mean keratometry were analyzed, and the refractive prediction error (PE) was calculated using different formulas. The study found that the AL difference in the operated eyes was significant when measured using the pseudophakic option but not with the aphakic option. However, the changes in the AL did not significantly affect the accuracy of the IOL power calculation, except for a systematic error in the optical biometer in the case of phakic eyes. A correction factor applied to the preoperative AL could eliminate this error without modifying the lens constantly.

Diabetic retinopathy (DR) is a serious eye condition that can have a significant impact on public health and vision. It is caused by the long-term effects of diabetes mellitus, which can result from resistance to insulin (hyperglycemia) in type 2 diabetes or autoimmune destruction of insulin-producing cells in type 1 diabetes. DR is a common late-stage symptom of diabetes and is a leading cause of blindness. Further studies have shown that the effective management of blood sugar levels can significantly reduce the risk of DR.

DR affects the microvascular system of the retina, causing pericyte loss, endothelial degradation, and eventually capillary permeability. This damage can occur without any early warning signs, which is why regular eye checkups are critical for early detection and treatment [1,3]. Up to 80% of individuals with diabetes for 20 years or more develop DR. The International Diabetes Federation predicts that the number of people living with diabetes will continue to rise, reaching 700 million by 2043. DR alters the blood vessels in the retina and progresses through five stages, each with specific clinical signs [3]. For example, microaneurysms are small lesions in blood vessels, while exudates are white or yellowish-white spots that are caused by protein leakage from microaneurysms. Increased fluid leakage into the retina leads to hemorrhages, which can ultimately cause permanent vision loss [4]. Retinal photography is a widely accepted screening tool for DR, with a manual interpretation having a better performance than in-person dilated eye examinations. Timely treatment is crucial in preventing permanent vision impairment, and regular eye checkups are recommended for those with long-term diabetes [5]. OCT plays a significant role as a tool in monitoring disease progression in DR. While DR is a serious condition, early detection and proper management can help reduce its impact on vision and improve the overall quality of life for those affected [6].

OCT is a multidisciplinary field that draws on research concerning lasers, optical sensors, and sparse signal processing, as well as image reconstruction, image classification, and medical image enhancement methods. The field of medical imaging, which has recently seen an expansion in ophthalmology with an emphasis on retinal imaging, is home to many deep learning (DL) applications. On the other hand, image analysis and diagnosis are not the main uses of DL in medicine [7,8,9]. The above methods can be used to evaluate a different data type of data such as clinical and demographic data. The goal of this work is to create a computer-aided diagnostic (CAD) that uses ML algorithms and OCT image data to automate DR diagnosis. Specifically, we propose an enhanced optical coherence tomography (EOCT) model based on a modified ResNet (50) pre-trained architecture and random forest algorithm using dual stochastic gradient descent (SGD) and Adam optimizers to improve the performance. The results of the experiments show that the EOCT-proposed model outperforms the compared works, including the spatial separable convolutions (SSC), VGG (16), and Inception v3 models, based on different metrics.

The structure of the remaining sections of this paper is as follows. Some of the most current studies in OCT classification and diagnosis are presented in Section 2. Section 3 presents the problem definition. Section 4 provides the experiments and their related results with comparisons to other related works. The conclusion of this work is presented in Section 5.

## 2. Enhanced Optical Coherence Tomography (EOCT) Model

**Figure 1:**
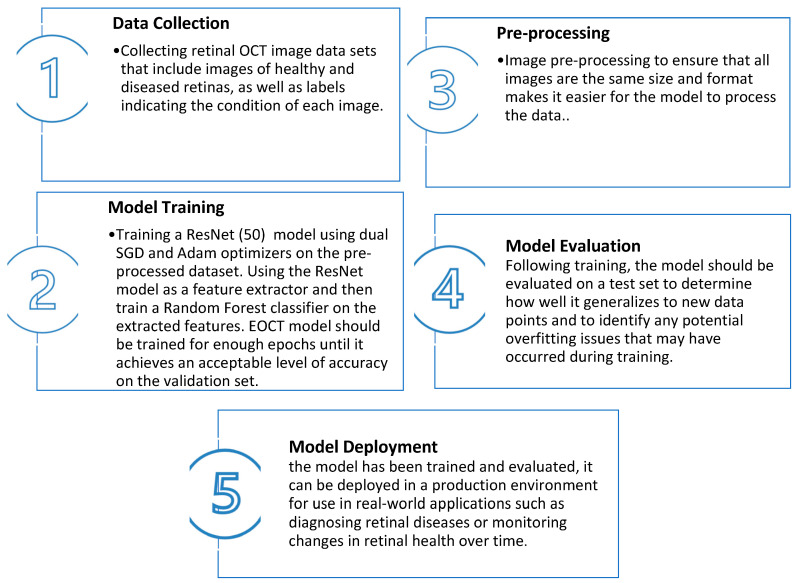
The proposed EOCT steps.

**Figure 2:**
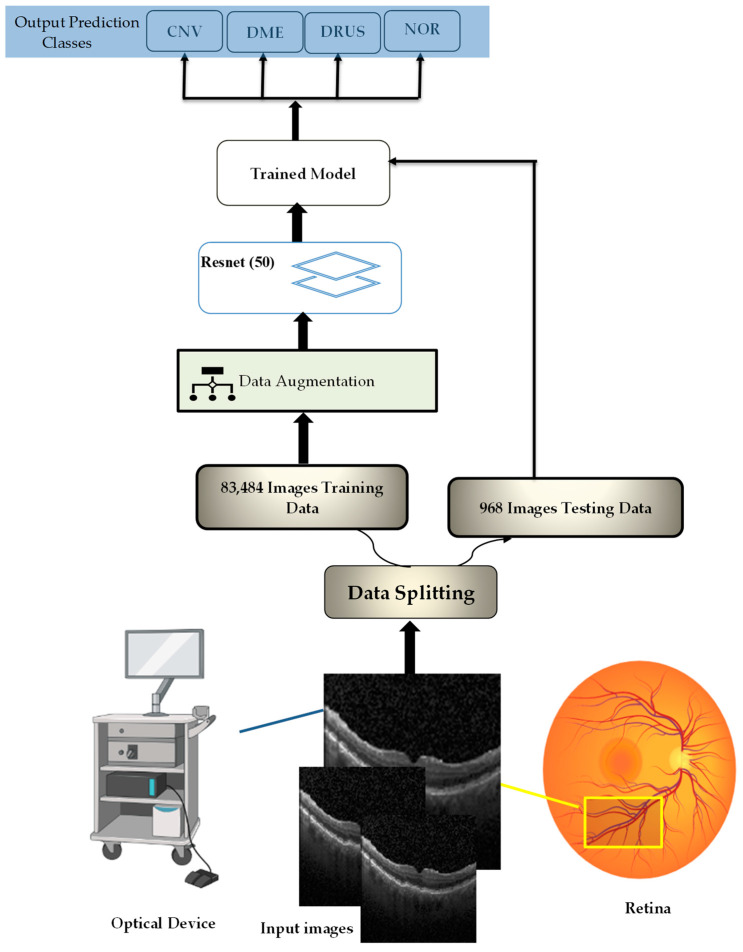
The illustration of the proposed EOCT.

This paper proposes an enhanced optical coherence tomography (EOCT) model for retinal OCT image classification based on a modified ResNet (50) pretrained architecture and random forest algorithm using dual SGD and Adam optimizers. The dual optimizers help in a faster convergence of the model, while the ResNet (50) architecture provides a better performance. This combination of an Adam optimizer and ResNet (50) architecture helps to achieve better results on retinal images that are captured from an OCT optical sensor. The illustration and overall steps of the proposed work of classifying OCT images are depicted in Figure 1 and Figure 2, respectively. The Retinal OCT Images dataset is a popular dataset that is used to detect various eye diseases [10].

Age-related macular degeneration (AMD) is a chronic and progressive disease of the macula, the central part of the retina that is responsible for sharp and detailed vision. It is one of the leading causes of visual impairment and blindness in people over 50 years old. The pathology of AMD is characterized by the accumulation of drusen, yellowish deposits of extracellular material, between the retinal pigment epithelium and Bruch’s membrane. There are two forms of AMD: dry (non-exudative) and wet (exudative). Wet AMD is caused by choroidal neovascularization (CNV), the growth of abnormal blood vessels from the choroid into the retina, which can leak blood and fluid and cause retinal damage.

Optical coherence tomography (OCT) is a non-invasive imaging technique that uses light waves to create high-resolution images of the retina. OCT pathological findings include CNV, diabetic macular edema (DME), drusen, and normal features. CNV appears as irregularly elevated or depressed retinal lesions with a hyperreflective border. DME is characterized by retinal thickening and fluid accumulation in the macula due to diabetes-induced damage to the retinal blood vessels. Drusen appears as round, discrete deposits with a hyperreflective center and hyporeflective halo. Normal OCT features show a clear and defined retinal structure.

The random forest algorithm is a robust ML algorithm that can be used to classify OCT images into four classes: choroidal neovascularization (CNV), deep margin elevation (DME), drusen, and normal. The algorithm works by constructing a multitude of decision trees from randomly selected subsets of the data. Each tree is then used to make a prediction on the class of an OCT image. The final prediction is made by taking the mode of all the individual tree predictions. The random forest algorithm has been shown to be effective at classifying OCT images with high accuracy and low false positive rates [11,12,13]. The consecutive steps can be written as follows: (i) begin by randomly selecting a subset of features from the OCT dataset; (ii) create multiple decision trees using the selected features; (iii) for each decision tree, randomly select a subset of training examples from the OCT dataset; (iv) train each decision tree using the selected training examples and features; (V) for each new example in the OCT dataset, make a prediction by having each decision tree make a prediction, and then take the majority vote of all predictions made by all decision trees; and (Vi) the final prediction is then assigned to one of the four classes (CNV, DME, drusen, or normal). ResNet (50) is a deep residual NN architecture that uses skip connections between layers to improve accuracy while reducing training time. It consists of 50 layers, which are used together to accurately classify images while reducing overfitting. When conducting the OCT image classification using ResNet-50, the output layer of the network will have a softmax activation function, which generates a probability distribution over the four classes. In comparison, SSC only consists of two separate convolutional layers, which are used together to reduce both the parameters count and computation time while still achieving good accuracy on the image classification task. Algorithm 1 presents the steps of the proposed architecture.

To validate the efficiency of our proposed work, we conducted a comparison of various types of CNNs. We illustrated the SSC architecture that has been proven effective and compared it with our proposed model. The SSC model can be used to reduce the number of parameters in a model, allowing for faster training times and better generalization; however, it cannot select all the features in an image, leading to a poorer performance than standard convolutions. VGG (16) is a DL architecture that is widely used for image classification tasks. It is composed of 16 layers, including 13 convolutional layers and 3 fully connected layers. VGG (16) requires a large amount of data for training, which can be difficult to obtain in some cases. It is composed of multiple inception modules that allow it to capture both local and global features in an image simultaneously. The Inception v3 model requires a large amount of data for training, which can be difficult to obtain in some cases; it uses skip connections, which allow it to capture both local and global features in an image simultaneously while reducing the number of parameters required for training compared with other architectures, such as VGG (16). ResNet (50) requires a large amount of data for training, which can be difficult to obtain in some cases.
**Algorithm 1:** Model Building AlgorithmInput:
○Training dataOutput:○Evaluated ModelSteps:
# Initialize ResNet (50) modelmodel = ResNet (50)# Define dual optimizers using SGD and Adamoptimizer_SGD = optim.SGD(model.parameters(), lr = 0.001)optimizer_Adam = optim.Adam(model.parameters(), lr = 0.001)# Load Retinal OCT Images datasetdataset = load_Retinal_OCT_Images()# Split dataset into train and test setstrain, test = split_dataset(dataset)# Train model using dual optimizers on train setfor epoch in range(num_epochs):# Train model with SGDfor batch in train:# Calculate gradients with SGDgradients = calculate_gradients(batch, optimizer_SGD)# Update weights with SGDupdate_weights(gradients, optimizer_SGD)# Train model with Adamfor batch in train:# Calculate gradients with Adamgradients = calculate_gradients(batch, optimizer_Adam)# Update weights with Adamupdate_weights(gradients, optimizer_Adam)# Evaluate model on test set after each epochevaluate (model, test)

### 2.1. Spatial Separable Convolutions (SSCs)

The SSC model is considered an NN layer that reduces the number of parameters and computations required to train the CNN. SSC consists of two separate layers: one for the spatial dimension and one for the depth dimension, which allows the network to learn more efficiently and with fewer parameters, resulting in faster training times and accuracy improvements. In a traditional convolutional layer, each filter is applied to all channels of the input feature map. It can decrease the number of computations and parameters required to train CNN, which can result in shorter training times and greater accuracy because fewer computations and parameters need to be learned [14]. SCC separates the learning of spatial features and channel-wise features; this makes sense because neighboring pixels in an image are often highly correlated, while different channels are typically independent [15].

The proposed approach achieves an optimal separation by using a principled approach to determine the internal number of groups and kernel sizes. The proposed approach achieves a complexity of O(n) and can achieve even lower complexity when the number of separated convolutions is not restricted. To maximize the performance with fewer parameters and computations, it can be challenging to decide which channels should be used for each filter. The SSC model has several main steps for the input feature map: (i) iteration over all channels in the feature map; (ii) application of a filter to the current channel; and (iii) storing of the output in an output feature map [14]. As SSC reduces the number of parameters that must be learned, it can aid in reducing overfitting.

### 2.2. VGG(16) Architecture

The VGG (16) architecture is a CNN model that was developed by the Visual Geometry Group at Oxford University. It differs from previous high-performing models in a few keyways. Firstly, it uses a 3 × 3 receptive field with a one-pixel stride, which is a departure from AlexNet’s 11 × 11 receptive field with a four-pixel stride. This design allows for more activation layers to work alongside the convolution layers, which improves decision functions and facilitates faster network convergence. Additionally, VGG (16)’s fully connected layers consist of 4096 neurons each.

**Figure 3:**
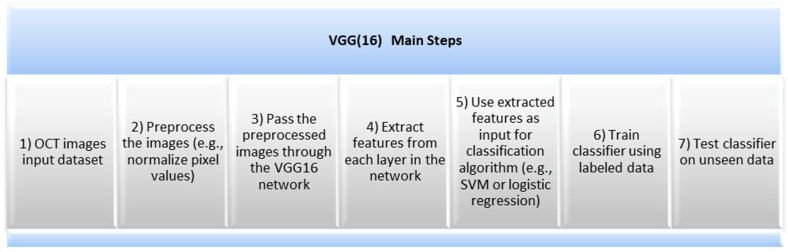
VGG (16) Main steps.

Secondly, VGG (16) uses a smaller convolutional filter to reduce overfitting during training. The optimal size was determined to be 3 × 3 as it allowed for the capturing of both horizontal and vertical information. Despite having a total of 138 million parameters, which is relatively high by today’s standards, VGG (16)’s simplicity was its main attraction. The architecture incorporates the most important convolution neural processes, and the process of training VGG (16) is like that of AlexNet. To avoid a vanishing gradient that can arise from the network’s depth, VGG uses mini batches [16]. VGG (16) architecture is designed to recognize patterns in images and classify them into different categories, as shown in Figure 3.

To extract information from the input image and subsequently categorize it into one of the predetermined classes, the VGG (16) model combines convolutional and fully connected layers. The precision, quickness, and scalability of VGG (16) are its benefits. It has been demonstrated that it performs better than other architectures on a variety of image classification tasks, including scene comprehension and object recognition. A further application is transferring learning, which enables users to modify the model for their own applications without having to start from zero.

Due to its intricacy, VGG (16) has the drawback of requiring a lot of data for training. Moreover, when running on a graphics processing unit or central processing unit with limited resources, it can be computationally expensive due to its depth.

### 2.3. Inception v3

Inception v3 is a CNN for image classification tasks that consists of a series of convolutional layers, pooling layers, and fully connected layers to extract features from the input image. The pooling layers are used to reduce the dimensionality of the feature maps. The fully connected layers are used to classify the input image into one of several classes. The model overcomes the overfitting problem by using an “inception module”, which consists of multiple parallel convolutional filters of different sizes and depths [17]. The Inception v3 model is an advanced and optimized version of the previous model. It is more efficient, has a deeper network, and is computationally less expensive while maintaining its speed. Several techniques were used to improve the model’s adaptation, i.e., factorization into smaller convolutions, spatial factorization into asymmetric convolutions, and the use of auxiliary classifiers as regularizers. The most significant modification to the Inception v3 model was the use of auxiliary classifiers to combat the vanishing gradient problem in very deep networks. Although they did not result in any improvement in the early stages of training, the model showed higher accuracy towards the end. Another modification was the spatial factorization into asymmetric convolutions, which are of the form n × 1, resulting in a relative gain of 28%. By factorizing larger convolutions into smaller ones, the model’s generous dimension reduction was further improved [18].

**Figure 4:**
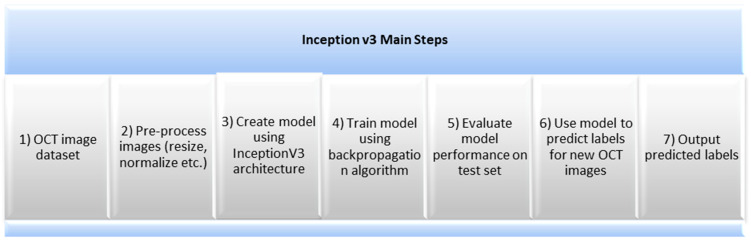
Inception v3 main steps.

These optimizations have made the Inception v3 model more efficient and effective and have helped it achieve better results in image classification tasks. Figure 4 shows the main steps for the Inception v3 pre-trained model.

The SSC model achieve can be used to increase the depth of a model without increasing the number of parameters, allowing for more complex models with fewer parameters and a small receptive field size; however, it can introduce artefacts into an image. VGG (16) is particularly challenging to implement on mobile devices as it needs a lot of processing and memory resources. As factorized convolutions minimize the number of parameters needed for training, Inception v3 is computationally efficient when compared with alternative architectures. Nevertheless, it needs a lot of data for training, which can be challenging in some circumstances.

## 3. Experimental and Results

Retinal OCT is a non-invasive imaging technique used to capture high-resolution cross-sectional images of the retina. OCT datasets are used to diagnose and monitor a variety of retinal diseases, such as age-related macular degeneration, diabetic retinopathy, and other retinal pathologies. OCT datasets typically consist of three-dimensional volumetric images that can be used to measure the thickness of the retina and identify any abnormalities. The data can also be used to track changes in the eye over time and help clinicians make more informed decisions about treatment options.

### Dataset

**Figure 5:**
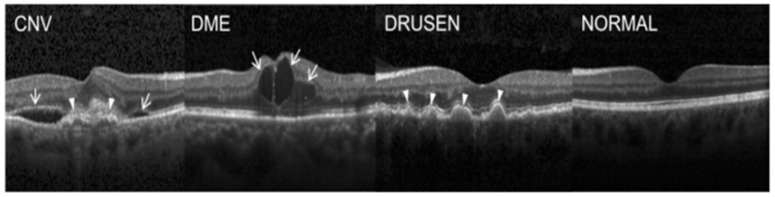
OCT dataset samples.

The Shiley Eye Institute at the University of California, San Diego, the California Retinal Research Foundation, and Medical Center Ophthalmology Associates all contribute OCT images from retrospective patient cohorts. An estimated 30 million OCT scans are performed annually, and it takes a substantial amount of time to analyze and interpret these images [19]. The dataset is divided into three folders (train, test, and validation) and comprises subfolders for each picture type (normal, CNV, DME, and drusen). It contains 84,495 X-ray images (JPEG) that are 224 × 224 in size for preprocessing. Among the 84,495 X-ray images (JPEG), the training sets including (CNV (37.2 K), DME (11.3 k), drusen (8616 files), normal (26.3 k)), validation (CNV (8), DME (8), drusen (8), normal (8)), and testing (CNV (242), DME (242), drusen (242), normal (242)) are shown in Figure 5. Spatial separable convolutions have been used in experimental analyses for retinal OCT to improve the accuracy of segmentation and classification of retinal layers. This technique involves breaking down a 2D convolution into two 1D convolutions, which can reduce the number of parameters and the computational complexity.

**Figure 6:**
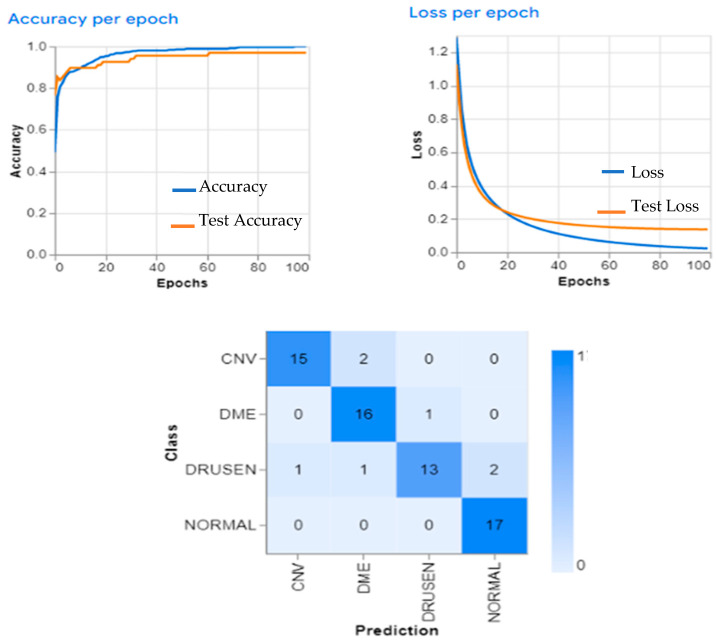
The EOCT proposed work learning curves.

**Figure 7:**
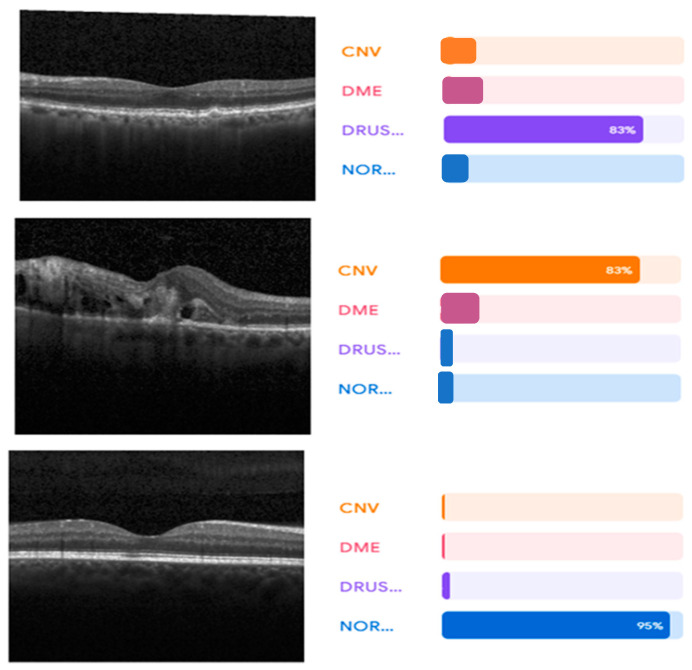
Testing visualization samples.

ResNet (50) and the dual (SGD, Adam) optimizers for the retinal OCT were tested on a dataset of 16,899 OCT images from healthy eyes, which was split into training and testing sets at a ratio of 80:20. The performance of this model was evaluated using the accuracy, precision, recall, F1-score, and AUC. The results showed that the ResNet (50) architecture with dual (SGD and Adam) optimizers achieved an accuracy of 97.47%, as shown in Figure 6 and Figure 7. The OCT dataset with (CNV, DME, drusen, normal) classes for the testing samples was analyzed using a variety of DL algorithms. The results showed that the best performing algorithm was the random forest algorithm; in terms of precision and recall scores, the random forest algorithm had the highest scores for all four classes: CNV (0.98), DME (0.97), drusen (0.96), and normal (0.99). Logistic regression had slightly lower precision and recall scores than the other two algorithms: CNV (0.93), DME (0.92), drusen (0.91), and normal (0.97).

**Figure 8:**
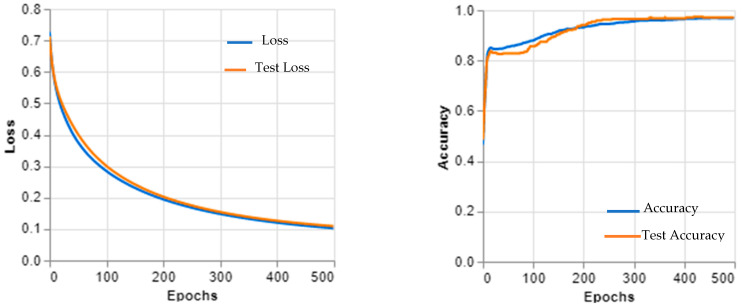
SSC architecture learning curve.

The accuracy of the SSC model is shown in Figure 8, demonstrating its effectiveness at classifying retinal layers. However, spatial separable convolutions have some limitations when applied to OCT images. The SCC model is not able to capture the fine details of the image due to its limited receptive field size. As a result, it cannot be able to accurately identify small features such as microaneurysms or drusen deposits in OCT images. This indicates that the model has a high degree of accuracy and can generalize well from the training data. Overall, these results demonstrate that the Retinal OCT dataset can be effectively used for image classification tasks with high accuracy.

**Figure 9:**
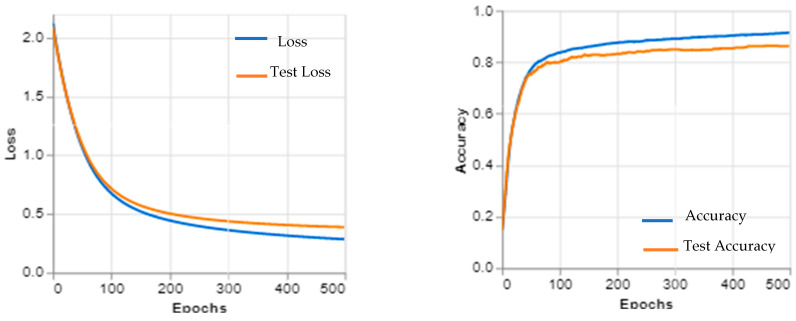
VGG (16) architecture learning curves.

**Figure 10:**
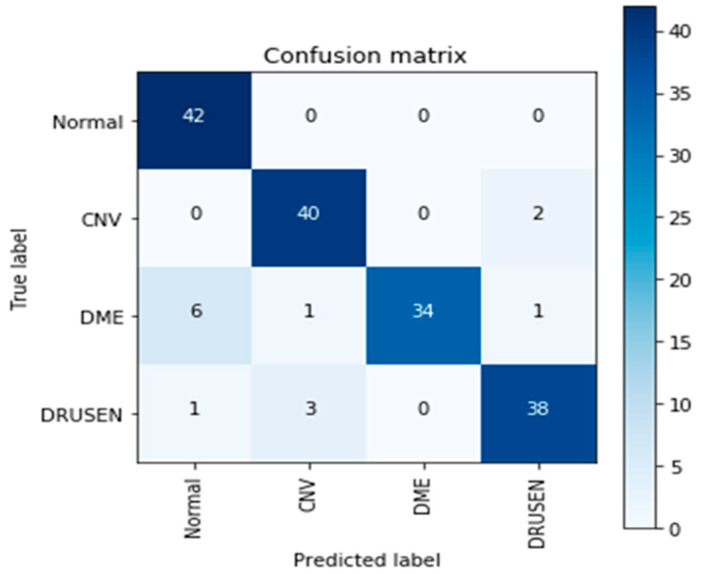
The confusion matrix of VGG (16).

VGG (16) uses a combination of high-resolution OCT imaging and advanced image processing algorithms to provide detailed information about the retinal layers. However, there are some limitations associated with VGG (16). First, it requires a highly trained technician to operate the OCT machine and interpret the images correctly. Second, it is expensive and is not widely available in many areas. Furthermore, it cannot detect certain types of retinal abnormalities, such as macular holes or epiretinal membranes. Therefore, other imaging techniques need to be used in conjunction with VGG (16) for a more comprehensive evaluation of the retina. Figure 9 and Figure 10 show the learning curves and the confusion matrix values, respectively.

This dataset was carefully curated to include images from both patients with and without retinal diseases to ensure that the model was able to accurately distinguish between the two. During the training process, the model was exposed to a wide range of OCT images and learned to recognize key features associated with retinal diseases. Once the training was complete, the model was tested on a separate test set to evaluate its accuracy in detecting retinal diseases. The results of this experiment showed that Inception v3 was highly effective at detecting retinal diseases, where it achieved an accuracy of 92%. This performance is comparable to other deep learning models used for this task and suggests that Inception v3 could be a useful tool for diagnosing and treating retinal diseases. However, it is important to note that Inception v3 does have some limitations when it comes to analyzing OCT images.

The Inception v3 architecture cannot learn all of the complex features necessary for an accurate diagnosis, and the size of the dataset used in this experiment was relatively small compared with other medical imaging tasks.

**Figure 11:**
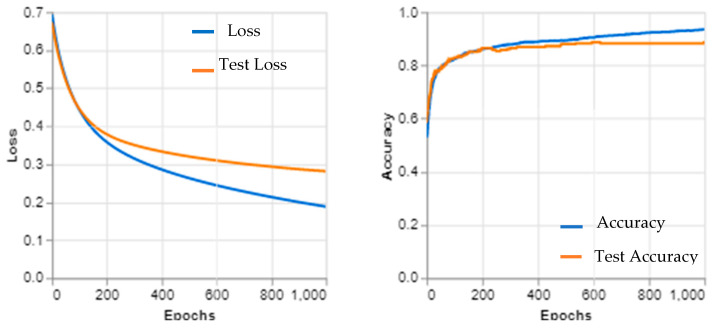
Inception v3 learning curves.

**Figure 12:**
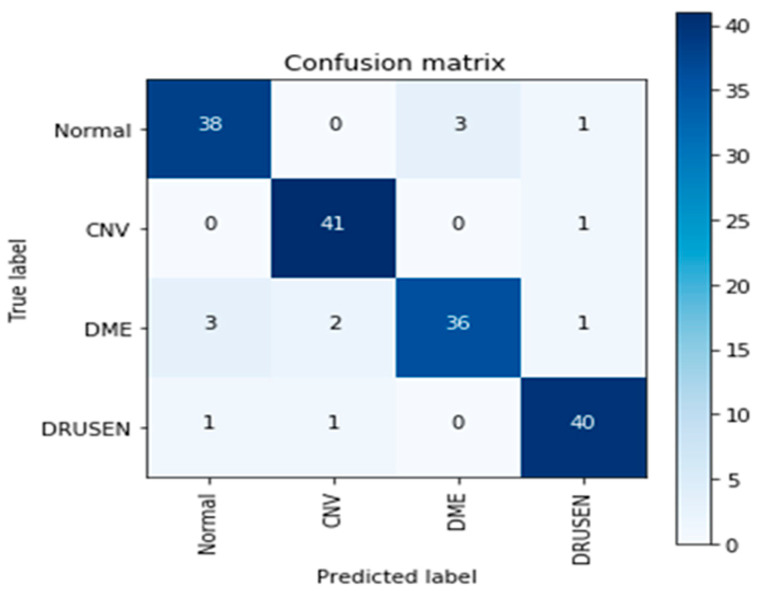
The confusion matrix of Inception v3.

These limitations highlight the need for continued research and development in the field of medical image analysis as well as ongoing efforts to improve the accuracy and generalizability of deep learning models like Inception v3. Figure 11 and Figure 12 show the learning curves and confusion matrix values, respectively.

In Table 1, FNR is the false negative rate, FDR is the false discovery rate, FPR is the false positive rate, NPV is the negative predictive value, and MCC is the Matthews correlation coefficient.

**Figure 13:**
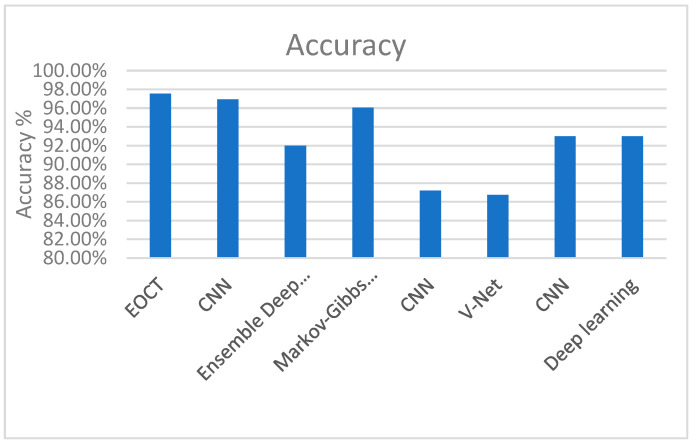
Comparing the proposed model and the related works.

A comparison between the proposed algorithm (EOCT) and some related works is illustrated in Table 2 and Figure 13.

From Table 2 and Figure 13, it is shown that the proposed EOCT outperforms the previous models. The results presented in this study suggest that the proposed enhanced optical coherence tomography (EOCT) model based on the modified ResNet (50) and random forest algorithms with dual SGD and Adam optimizers outperforms previous models with respect to accurately classifying OCT images into four classes: CNV, DME, drusen, and normal. These findings have important implications for the diagnosis, classification, and monitoring of various retinal diseases, such as age-related macular degeneration and diabetic retinopathy.

One limitation of this study is the need for a large and diverse dataset to train the algorithms, which cannot always be available. This limitation highlights the importance of obtaining large and diverse datasets to improve the generalizability and accuracy of the proposed algorithms for medical image classification tasks. Additionally, the accuracy of the algorithms varies depending on the specific disease being classified and the quality of the OCT images. Therefore, further studies with larger and more diverse datasets could improve the generalizability and accuracy of the proposed algorithms for medical image classification tasks.

The proposed EOCT model based on the modified ResNet (50) and random forest algorithms with dual SGD and Adam optimizers showed promising results for accurately classifying OCT images into four classes: CNV, DME, drusen, and normal. These results demonstrate the potential of these algorithms for accurately classifying OCT images for medical diagnosis, which can potentially provide a more efficient and objective way of analyzing OCT images. Further studies with larger and more diverse datasets could improve the generalizability and accuracy of the proposed algorithms for medical image classification tasks. The authors in [27] are acknowledged for their research on BRVO and CRVO, which are major causes of visual impairment worldwide, particularly among the elderly. In another study, motor and cognitive impairments in PSP were evaluated using the PSP rating scale and Montreal Cognitive Assessment Battery (MoCA), respectively. The exclusion criteria included eyes with inadequate image quality or comorbid conditions. SD-OCT measures were utilized to compare the PSP and control groups, and the correlations were examined between the retinal layer thicknesses and disease severity. The results indicated that PSP patients had a statistically significant reduction in the thickness of the inner retinal layer (IRL), ganglion cell layer (GCL), inner plexiform layer (IPL), and outer plexiform layer (OPL) when compared with healthy controls [4].

In the current study, the proposed enhanced optical EOCT model has been developed based on the modified ResNet (50) and random forest algorithms using dual SGD and Adam optimizers. The results of the experimentation showed promising results for accurately classifying OCT images into four classes: CNV, DME, drusen, and normal. This indicates that the proposed algorithms can potentially provide a more efficient and objective way of analyzing OCT images for medical diagnosis [28].

The high accuracy and precision achieved by the proposed algorithms demonstrate the potential of these algorithms for accurately classifying OCT images for medical diagnosis. The random forest algorithm achieved an impressive accuracy of 95.1% and precision of 96.8%, while the ResNet (50) with an Adam optimizer achieved an even higher accuracy of 97.56%.

One potential limitation of this study is the need for a larger and more diverse dataset to train the algorithms, which may not always be readily available. The accuracy of the algorithms may also vary depending on the specific disease being classified and the quality of the OCT images. Consequently, further studies with larger and more diversified datasets could enhance the generalizability and precision of the proposed algorithms for medical image classification tasks.

In conclusion, the proposed EOCT model based on the modified ResNet (50) and random forest algorithms with dual SGD and Adam optimizers shows promising results with respect to accurately classifying OCT images into different classes. This indicates that the proposed algorithms have the potential to provide a more efficient and objective way of analyzing OCT images for medical diagnosis.

## 4. Discussion

Image processing of retinal images is a popular topic in the scientific community. There are numerous methods for automatically categorizing the severity of DR. A group of machine learning (ML) approaches known as DL allow for computational models that are made up of many processing layers to learn how to represent the input. One of the most widely applied methods for retinal imaging and image classification tasks is the convolutional neural network (CNN) [29,30,31]. In general, DL has demonstrated its superiority over conventional methods by offering a sizable workforce, substantial financial resources, and obtaining high accuracy in several areas [10,20,31,32]. DL has been used to examine key eye illnesses including DR, and glaucoma age-related macular degeneration, which either rely on standard recommendations or demand a long-term follow-up. Thanks to its great diagnostic performance for recognizing diverse pathological states [14,15,17,18,33,34,35]. Due to the lack of studies on OCT image processing and the fact that OCT angiography (OCTA) is a relatively new modality, most earlier studies have used color fundus images for the segmentation of retinal blood vessels.

Shen et al. [35] presented a structure-oriented transformer (SOT) model for grading retinal diseases from OCT images. Their model used a transformer-based architecture and structure-oriented attention mechanism to capture the structural information of the retina and improve the accuracy of the disease grading. The results showed that the SOT model outperformed other models in terms of accuracy and could effectively grade a range of retinal diseases. Heisler et al. [21] built a neural network (NN) from single data types and conducted fine-tuning based on some DL pre-trained architectures to diagnose DR from OCTA and OCT images. The results showed that the ensemble networks constructed with four fine-tuned VGG (19) architectures outperformed the other DL architectures and yielded accuracies of roughly 0.90 and 0.92 respectively. Diao et al. [20] presented a method for classifying and segmenting OCT images for age-related macular degeneration (AMD) using dual guidance networks. Their proposed method uses a two-stage process, where the first stage involves classifying OCT images into healthy or AMD categories and the second stage involves segmenting the AMD regions within the OCT images. The dual guidance network approach utilizes both pixel-level and feature-level guidance to improve the accuracy of the classification and segmentation. Eladawi et al. [22] proposed a CAD system for DR diagnosis using OCT that incorporates retinal vascular (RV) segmentation, image-derived markers, and an SVM-based classification. This system uses a joint Markov–Gibbs random field (MGRF) model based on a stochastic technique to describe the formation of blood vessels at various levels in diabetic and normal instances. Their method can identify a variety of illnesses of the choroid and retina based on the biomarkers collected from OCT scans. The results showed that the image with the boosted stage had achieved a differential scanning calorimetry (DSC) of 96.04%. Transfer learning was used by Le et al. [23] to automate OCT categorization using CNN and VGG (16). A dataset consisting of 131 observations was used for training and cross-validation. The results showed that the CNN architecture delivered the best results after retraining the final nine layers, where the cross-validation specificity, sensitivity, and accuracy of the retrained classifier achieved results of 90.82%, 83.7%, and 87.27%, respectively, for differentiating between DR, non-DR, and healthy eyes. Elgafi et al. [36] proposed a system for identifying diabetic retinopathy that could affect the eyes using a 3D OCT technique. They first segmented the 3D OCT retina layers, followed by extracting two features from the layers to be fused for training and testing using an NN classifier. They concluded that their work, based on 188 cases, outperformed the compared DL algorithms, in which the model achieved a 96.81% accuracy. Table 3 provides a comparison of some related studies using OCT. A DL-based framework for automatic artery/vein (AV) categorization in OCTA was proposed by Alam et al. [24]. The authors’ results showed that the AV-Net had an F1-score of 82.81%, a mean IOU of 70.72%, and an average accuracy of roughly 86.75%. Their study had certain limitations because there were significant areas of misclassification, including at vessel cross-sites. For OCTA projection images utilizing the two types of FOVs, Diáz et al. [25] proposed a model using a range of morphological operators; the technique used initially locates the region and then extracts its accurate outline using a combination of image processing algorithms. This method achieved an accuracy of 0.93 for diabetic OCTA images and approximately 0.93 for healthy subjects (0.82). A wide-field swept source OCTA method was employed by Kim et al. in [26], where the authors proposed semiautomated diagnostics for microvascular parameters for grading the severity of DR using a variety of viewpoints. Proliferative DR (PDR) and diabetes without retinopathy were the five categories used in their study to group 235 diabetic eyes. Ong et al. [37] proposed a model based on deep capillary plexus (DCP)-skeletonized vasculature length to segment and threshold the OCTA slabs’ DCP segments based on the length of the DCP skeletonized vessels. After taking imaging quality into account, the vascular length density (VLD) and all three capillary layers were compared between every DR severity group. The experiment results showed that the AUC values ranged from 0.731 to 0.752, while the specificity ranged from 57.1% to 64.3% respectively. The significant racial disparities between study groups and the DCP’s lack of power are two flaws in the study that have made it harder to detect actual changes in DCP characteristics across groups. Hamwood et al. [38] demonstrated that modifying specific elements of the CNN network layout can also greatly enhance the segmentation outcomes. The number of classes (i.e., boundaries) used to train the CNN can affect how well the approach performs; the presence of identical image attributes between classes, which can lead to false positives, makes the method perform worse when fewer classes are used for training. Elsharkawy et al. [39] presented a review and comparative analysis of four imaging modalities for the diagnosis of DR, such as OCT used with ML and DL, which included a large number of detailed images of the retina. The authors also focused on how these modalities are combined with clinical information and demographic data to increase the performance of automatically diagnosing and grading DR. Instead of learning unreferenced functions, He et al. [31] explicitly redesigned the layers to learn residual functions with reference to the layer inputs. The authors offered in-depth empirical proof that these residual networks are simpler to optimize and can improve accuracy over far more depth. The authors evaluated the residual nets using the ImageNet dataset with a depth of up to 152 layers, which is 8 times deeper than that of VGG nets but still has a lower complexity. On the ImageNet test set, an ensemble of these residual nets achieved a 3.57% error.

However, the majority of the earlier methods described in the literature have a number of limitations, such as: (i) current discriminative methods have insufficient features to adequately describe the OCTA problem; resulting in lower accuracies; (ii) the computationally expensive registration chores being a problem for current generating approaches. Additionally, the atlas does not accurately reflect the population of images; and (iii) the computationally high cost of training the CNN layers being a drawback of current DL techniques. Additionally, choosing the optimal number of layers and neurons for each layer remains an unsolved scientific issue.

## 5. Conclusions

In conclusion, retinal optical coherence tomography (OCT) has become an essential tool for diagnosing and monitoring various retinal diseases, including age-related macular degeneration and diabetic retinopathy. Although the use of OCT imaging has improved our understanding of ocular diseases, it still suffers from a subjective and time-consuming qualitative evaluation. In this study, we proposed an enhanced EOCT model that utilizes modified ResNet (50) and random forest algorithms with dual SGD and Adam optimizers to classify OCT images into four categories: CNV, DME, drusen, and normal. Our proposed EOCT model has the potential to provide a more efficient and objective way of analyzing OCT images for medical diagnoses. The experiments demonstrated that our proposed model achieved promising results for accurately classifying OCT images, with the random forest algorithm achieving an impressive accuracy of 97.47%.

However, our study has some limitations. We did not consider the analysis of choroidal features through enhanced depth imaging (EDI) OCT images, which could be important for understanding systemic pathologies. Furthermore, the accuracy of the algorithms varies depending on the specific disease being classified and the quality of the OCT images. Additionally, a large and diverse dataset is required to train the algorithms, which may not always be available. Therefore, future studies with larger and more diverse datasets could improve the generalizability and accuracy of the proposed algorithms for medical image classification tasks.

## Figures and Tables

**Table 1:** The results of the applied ResNet (50) and random forest algorithm.

Model	Sensitivity	Specificity	Precision	NPV	FPR	FDR	FNR	Accuracy	F1-Score	MCC
EOCT	0.9836	0.9615	0.9740	0.9756	0.0385	0.0260	0.0164	0.9747	0.9788	0.9474

**Table 2:** EOCT vs. different related works.

Year	Reference	Model	Dataset	Data Size	Accuracy
2023	The proposed work	EOCT	OCT2017	84,495 X-ray images	97.47%
2023	Diao et al. [20]	CNN	OCT2017	_	96.93%
2020	Heisler et al. [21]	Ensemble Deep Learning	OCT2017	_	92%
2017	Eladawi et al. [22]	Markov–Gibbs Random Field	OCT2017	_	96.04%
2020	Le et al. [23]	CNN	OCT2017	_	87.2%
2020	Alam et al. [24].	V-Net	OCT2017	_	86.75%
2019	Dáz et al. [25]	CNN	OCT2017	_	93%
2021	Kim et al. [26]	Deep Learning	OCT2017	_	93%

**Table 3:** A brief comparison of related works.

Year	Reference	Model	Task	Dataset	Evaluation Metrics (%)
2023	Diao et al. [20]	CNN	Retinal OCT Disease Classification	OCT2017	ACC = 96.93
2018	Shen et al. [35]	Structure-Oriented Transformer	Retinal OCT Disease Classification		N/A
2020	Heisler et al. [21]	Ensemble Deep Learning	Retinal OCT Disease Classification		ACC = 92
2017	Eladawi et al. [22]	Markov–Gibbs Random Field	Retinal OCT Disease segmentation		DSC = 96.04%.
2020	Le et al. [23]	CNN	Retinal OCT Disease classification		ACC = 87.2
2020	Alam et al. [24]	V-Net	Retinal OCT Disease classification		ACC = 86.75
2019	Diáz et al. [25]	CNN	Retinal OCT Disease classification		ACC = 93
2021	Kim et al. [26]	Deep learning	Retinal OCT Disease Classification		ACC = 0.93
2020	Ong et al. [37]	Deep Capillary Plexus (DCP)	Retinal OCT Disease Classification		Sensitivity = 83.3%
2018	Hamwood et al. [38]	CNN	Retinal OCT Disease Classification		N/A
2016	He et al. [31]	OCT Disease Classification	Image Classification		ACC = 86.65

## Data Availability

https://www.kaggle.com/datasets/paultimothymooney/kermany2018 (25 January 2023).
